# Melatonin regulates gene expressions through activating auxin synthesis and signaling pathways

**DOI:** 10.3389/fpls.2022.1057993

**Published:** 2022-12-13

**Authors:** Wei Wei, Jian-Jun Tao, Cui-Cui Yin, Shou-Yi Chen, Jin-Song Zhang, Wan-Ke Zhang

**Affiliations:** ^1^ State Key Lab of Plant Genomics, Institute of Genetics and Developmental Biology, Innovation Academy for Seed Design, Chinese Academy of Sciences, Beijing, China; ^2^ College of Advanced Agricultural Sciences, University of Chinese Academy of Sciences, Beijing, China

**Keywords:** melatonin, auxin, soybean, transcriptome, time series analysis

## Abstract

**Background:**

Both melatonin and indole-3-acetic acid (IAA) are derived from tryptophan. And the most interesting and unsolved puzzle in melatonin research is that what is the relationship between melatonin and auxin?

**Methods:**

In this study, we performed transcriptome analysis with a time series method to disclose the connection of the two metabolites in soybean.

**Results:**

Our results reveal that melatonin and IAA treatments cause substantial overlaps in gene expression changes. Common genes of melatonin and IAA treatments could be sorted into clusters with very similar expression tendency. A KEGG assay showed that exogenous applied melatonin enriched differentially expressed genes in auxin biosynthesis and signaling pathways. For details, melatonin up-regulates several *YUCCA* genes which participate in auxin biosynthesis; melatonin also enhances expression levels of auxin receptor coding genes, such as *TIR1*, *AFB3* and *AFB5*; dozens of genes involved in auxin transport, such as *AUXI* and *PIN*, are regulated by melatonin similarly as by auxin; auxin-responsive genes, such as *IAA*, *ARF*, *GH3* and *SAUR*-like genes, intensively respond to melatonin as well as to auxin. A DR5 promoter mediated GUS staining assay showed that low concentration of melatonin could induce auxin biosynthesis in a dosage manner, whereas high concentration of melatonin would eliminate such effect. At last, gene ontology (GO) analysis suggests that melatonin treatment has similar characteristics as auxin treatment in many processes. However, the two molecules still keep their own features respectively. For example, melatonin takes part in stress responses, while IAA treatment enriches the GO terms that related to cell growth.

**Conclusion:**

Taken together, exogenous applied melatonin, if not exceeds the appropriate concentration, could promote auxin responses range from biosynthesis to signaling transduction. Thus, our research is a key part to explain the auxin-like roles of melatonin in regulating plant growth.

## Introduction

Melatonin (N-acetyl-5-methoxy-tryptamine), a well-known bioactive molecule, was first found in bovine pineal glands (Lerner et al., 1960). In 1995, several research groups reported that melatonin also existed in plants (Dubbels et al., 1995; Hattori et al., 1995; Kolar and Machackova, 2005). From then on, the important roles of melatonin in higher plants have been revealed. The most familiar function of melatonin is to enhance biotic/abiotic stress tolerance of plants. As a powerful antioxidant, melatonin protects plants from oxidative damage (Tan et al., 2012; Arnao and Hernandez-Ruiz, 2019). More and more studies showed that the protection function of melatonin was ubiquitous in plants (Park et al., 2013; Zhang et al., 2015; Zhao et al., 2016; Moustafa-Farag et al., 2019).

What makes melatonin even more valuable is that it could also promote plant growth and even increase crop yield (Hernandez-Ruiz et al., 2005; Tan et al., 2012; Wei et al., 2015). In 2007, a pioneering study in lupin (*Lupinus albus L*.) connected melatonin with IAA, the most natural forms of auxin, in regulating root development. Both melatonin and IAA significantly increased growth of adventitious and lateral roots (Arnao and Hernandez-Ruiz, 2007). After that, the auxin-like functions of melatonin have been noticed by researchers and verified in other plants (Sarropoulou et al., 2012; Koyama et al., 2013). What roles does melatonin play in growth regulation? Does it have something to do with auxin? Some groups claimed that exogenous melatonin enhanced IAA amount (Chen et al., 2009; Wen et al., 2016); some believed that melatonin regulatory pathway was independent of auxin signaling (Pelagio-Flores et al., 2012; Koyama et al., 2013); other researchers even discovered repressive effect of melatonin on auxin biosynthesis (Wang et al., 2014; Wang et al., 2016). Though the answers are controversial, this topic is still fascinating in melatonin research. And we believe that using high throughput technology, we could analyze tens of thousands of genes that regulated by melatonin or IAA treatment. From the whole gene level, maybe we would tell the difference between melatonin and auxin in soybean.

To solve the above problem, the most crucial point is to tell exogenous melatonin from endogenous melatonin. It seems that manipulating endogenous melatonin biosynthesis using transgenic method causes consistent results, which could be summarized as enhancing melatonin amount but decreasing IAA amount (Wang et al., 2014; Zuo et al., 2014). So the controversy lies in the exogenous melatonin treatment (**Table S1**) (Arnao and Hernandez-Ruiz, 2018). The second important thing is to capture gene expression changes as many as possible. Treatment with a single time point would inevitably miss quite a lot of information, since expressing patterns of genes are various. Based on the above analysis, we designed a time series experiment. Finally, we got detailed and convincing results showing the impartible but also competitive relationship between melatonin and auxin in soybean.

## Materials and methods

### Plant materials and growth conditions

Soybean seeds (*Glycine max*, SuiNong 28, SN28) were sowed in pots filled with vermiculite and soil in a ratio of 1:1. Greenhouse for soybean growth and treatment were set to 28°C in the daytime and 16°C at night with a photoperiod of 16 h: 8 h (day: night).

### Melatonin or auxin treatment

Melatonin and auxin treatments were performed when soybean seedlings grew to V1 stage. Melatonin and IAA solutions were fertilized to the soil with the final concentration of 20 μM and 1 μM respectively. Each treatment contained 5 time points, such as 0, 1, 3, 6 and 12 h. For quantitative RT-PCR analysis, an independent experiment was performed. Melatonin and IAA treatments were repeated with the same concentration and time points. Besides, a mock treatment was also introduced, in which soybean seedlings were supplied with required water and mock samples were cut for RNA extraction at the same time points as melatonin and IAA treatments.

### RNA extracting and sequencing

Total RNA of soybean leaf and root was extracted respectively, using TRNzol Reagent (TIANGEN company). RNA sequencing was performed using an Illumina HiSeq instrument. Clean reads were mapped to soybean genome (https://phytozome.jgi.doe.gov/) using TopHat software (http://ccb.jhu.edu/software/tophat/) and differentially expressed genes were analyzed by cufflinks software (http://cole-trapnell-lab.github.io/cufflinks/).

### Advanced data analysis

Differentially expressed genes (DEGs) were defined as genes that had a two-fold increase or a 50% decrease in a certain time point compared to 0 h. The following analysis was performed using these DEGs. Venn diagram was performed using an online tool (http://bioinfogp.cnb.csic.es/tools/venny/index.html). Time series analysis was performed using R software of maSigPro. Bubble heat-map was drawn using R software of ggplot2. KEGG analysis was performed by KEGG mapper (https://www.genome.jp/kegg/tool/map_pathway2.html). Gene ontology analysis was performed by agriGO, using Z-score and PAGE methods (Parametric Analysis of Gene Set Enrichment) (Tian et al., 2017). A Pearson correlation was performed to establish co-expression network of DEGs in auxin biosynthesis and signaling pathways. The co-expression network was visualized by Cytoscape software.

### *Agrobacterium rhizogenes*-mediated transformation

The *A. rhizogenesstrain* K599 harboring DR5-*GUS* gene was injected to the hypocotyls of 5-day-old soybean seedlings, which was a position about 2 cm above soil surface. After injection, the seedlings were kept in high humidity for about 2 weeks, until hairy roots generated from the wound. The main roots were cut and the seedlings with transgenic hairy roots were moved to water. After a 5-day recovery, the seedlings were treated with different concentrations of melatonin and auxin for 24 h. After treatment, the hairy roots were cut for the following GUS staining. For long time auxin treatment, the more stable chemical, naphthylacetic acid (NAA), was used instead of IAA.

### GUS staining

GUS stocking solutions: 0.2 M NaH_2_PO_4_ (Stock A), 0.2 M Na_2_HPO_4_ (Stock B), 100 mM X-Gluc stock (dissolved with DMF or DMSO). For 10 ml of working solution, add 3.9 ml of Stock A, 6.1 ml of Stock B and 200 μl of X-Gluc stock. Put DR5:*GUS* transgenic hairy roots in working solution and keep it under 37°C for 6 h or room temperature for 12 h. After staining, GUS working solution was washed away with 70% ethanol; hairy roots were photographed under microscope. GUS photos were measured using ImageJ software to determine GUS intensity. For each concentration of treatment, three replications were measured.

## Results

### Common regulation of melatonin and IAA treatments in gene expression

**Figure 1:**
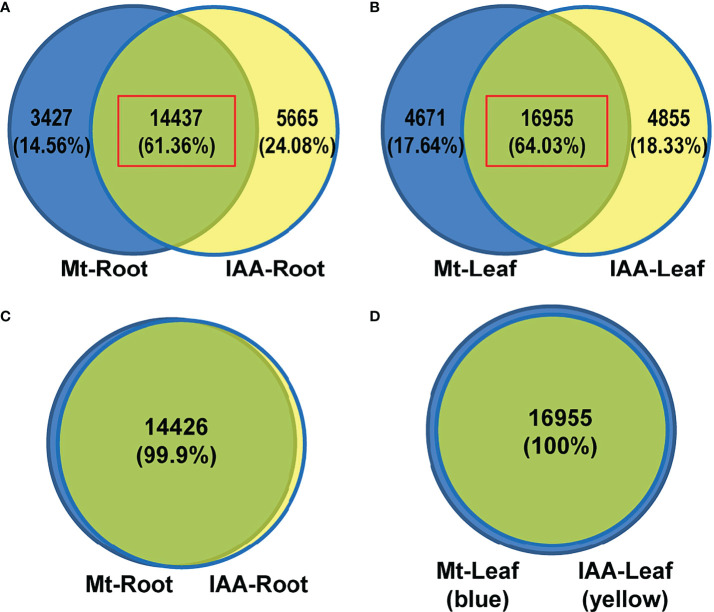
DEG analysis of Melatonin (Mt) or IAA treated transcriptome data. **(A)** Venn diagram of DEGs in roots. **(B)** Venn diagram of DEGs in leaves. **(C)** A venn diagram results from the time series analysis in roots. The 14437 common genes of melatonin- and auxin-treated roots, red box in **(A)**, were analyzed using a time series assay and only 11 of them were not clustered. **(D)** A venn diagram results from the time series analysis in leaves. The 16955 common genes of melatonin- and auxin-treated leaves, red box in **(B)**, were analyzed using a time series assay and all of them are able to be clustered.

To investigate the relationship between melatonin and auxin, soybean seedlings were treated with melatonin or IAA (1 μM) in a time series experiment. The auxin-like phenotype of melatonin appeared at the concentration of 10 μM in another fabaceae plant lupin, and such promotive effect was observed in soybean when seeds were coated with 50 μM melatonin using seed-coating-reagent (the actual concentration must be lower than 50 μM after sowing) (Arnao and Hernandez-Ruiz, 2007; Wei et al., 2015). Thus, we supposed that the appropriate concentration of melatonin treatment should be between 10 and 50 μM, and we finally decided to use the concentration of 20 μM in this treatment. Genes that had a two-fold increase or a 50% decrease in at least one time point against untreated samples (0 h) were defined as differentially expressed genes (DEGs). Root samples had a combined (melatonin treatment plus IAA treatment) number of 23529 DEGs, while leaf samples had a combined number of 26481 DEGs (**Figure S1**). Venn diagrams showed the distribution of these DEGs in melatonin (Mt) or IAA treatment. It is noteworthy that melatonin samples and IAA samples had very large overlap, which is about 61% (14437 common genes) in roots and 64% (16955 common genes) in leaves, suggesting that the connection of melatonin and auxin was very close (**Figures 1A, B**).

**Figure 2:**
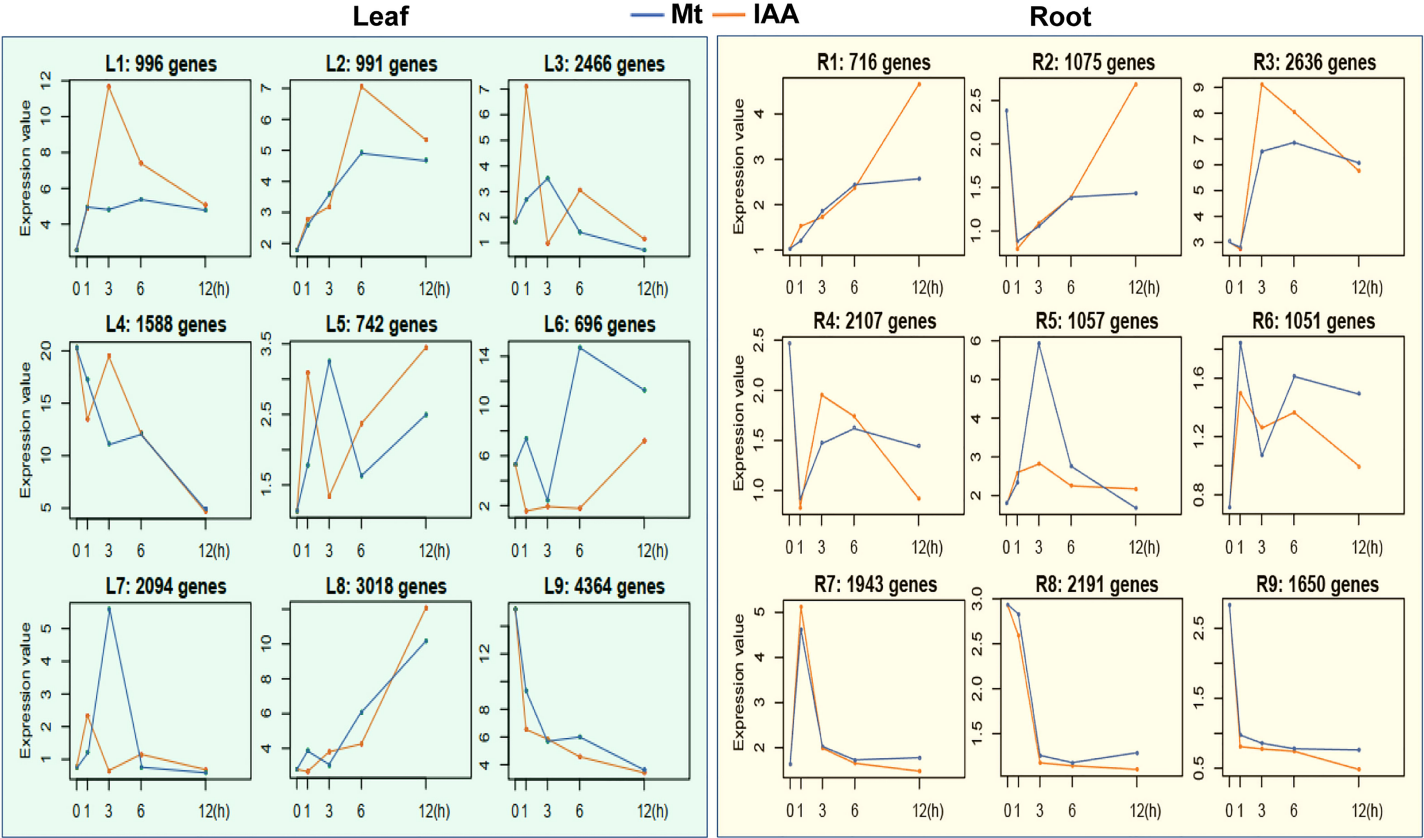
Time series analysis of DEGs in melatonin or auxin treated soybean seedlings. Common genes of melatonin (Mt) and auxin treatment in soybean seedlings were analyzed using a time series method. Each box indicates a cluster of genes. Blue lines indicate median profiles of the melatonin-responded genes, while orange lines indicate median profiles of auxin-responded genes.

To disclose expression tendencies of these common DEGs, we performed a time series analysis using common DEGs of melatonin and IAA treatments. Before the common DEGs were grouped into their belonging clusters, venn diagrams gave us a brief look that the DEGs were classified very well. Astonishingly, 100% of DEGs in leaf samples were successfully grouped; only 11 DEGs in IAA-treated roots could not be grouped (**Figures 1C, D**). Time series assay demonstrated that the common DEGs were classified into 9 clusters in root and leaf respectively, according to their expression patterns (**Table S2** and **S3**). In roots, three clusters of genes, R1, R3 and R6, were regulated more strongly by IAA than by melatonin, while the R4 cluster showed the opposite effect (**Figure 2**, right part). Just like in roots, genes in L1, L2 and L7 were induced to higher peaks by IAA than by melatonin, while L4 showed the opposite effect. The clusters of L2, L5, L6 and L9 must be mentioned, because they revealed different response speed between melatonin and IAA treatments. Take cluster L2 and L5 for examples, IAA samples reached their peaks in 1^st^ hour, while melatonin samples reached their peaks in 3^rd^ hour with a slower speed. Besides, melatonin samples in cluster L2 reached the peaks lower than that of IAA samples. Cluster L9 showed an effect of decreasing situation, in which IAA samples dropped to the valley faster than melatonin samples. The most complicated case is cluster L6. In 1^st^ and 3^rd^ hour, melatonin and IAA samples showed reverse effect, but in the long run, the expression patterns could be summarized. Expression levels of genes in cluster L6 dropped to the valleys in the beginning and then reached to the peaks, but finally they would fall again (**Figure 2**, left part). When dealing with leaf samples, long-term transport of molecules needs to be considered, and the above four clusters just showed us that time difference existed in this process. All of these clusters strongly support the idea that exogenous melatonin and IAA treatments may have a lot in common when they are applied to soybean seedlings, because their patterns perfectly matched each other.

### KEGG analysis and co-expression network reveals the effect of melatonin on auxin pathway

**Figure 3:**
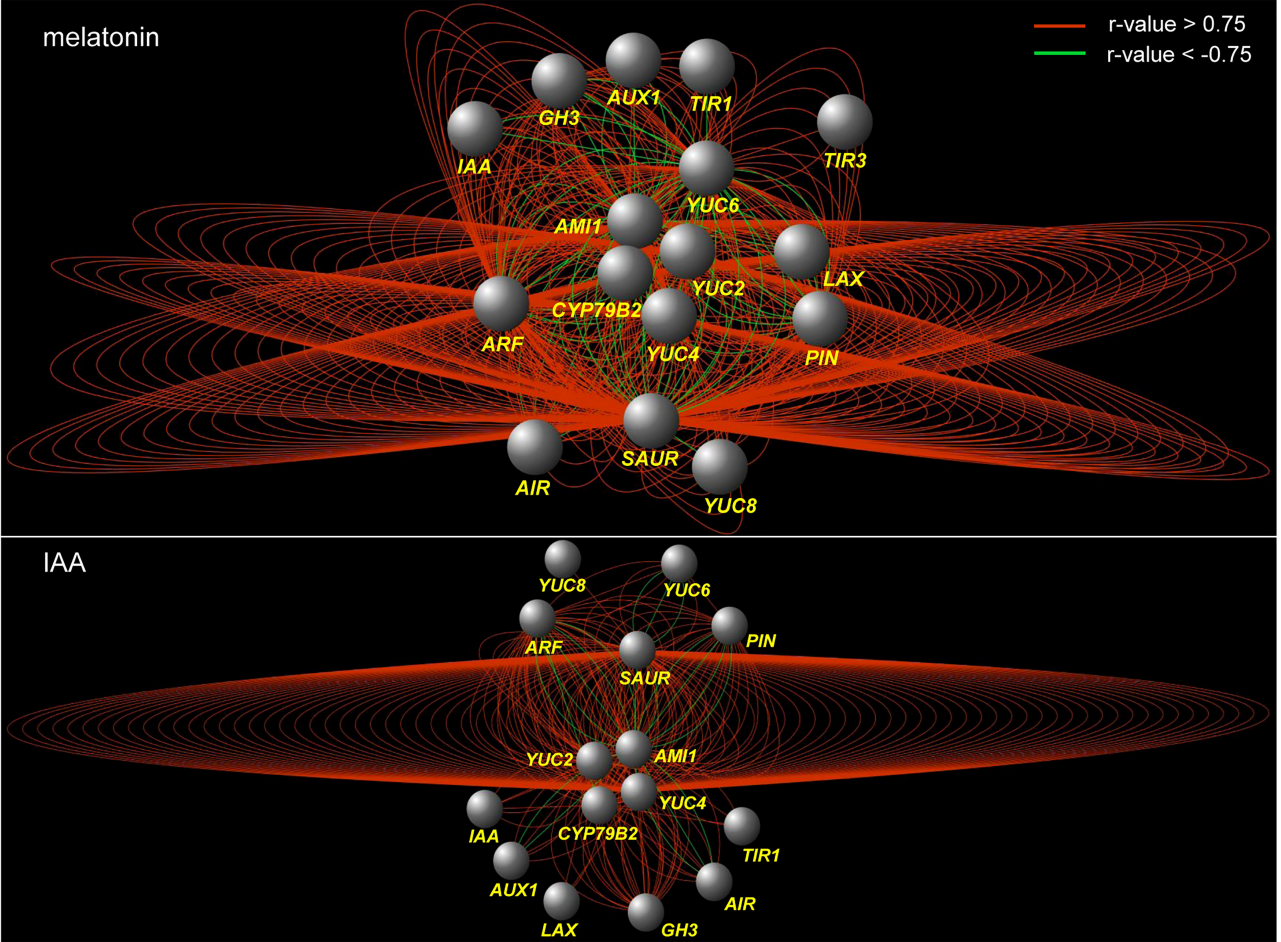
Co-expression network of the genes that are related to auxin biosynthesis and signaling pathways under melatonin and IAA treatments. The red edges indicate positive correlation (r > 0.75, FDR < 0.01), whereas the green edges indicate negative correlation (r < -0.75, FDR < 0.01). The correlation was calculated with ‘pearson’ method. The nodes indicate groups of genes which were listed in **Table S4**.

To disclose the effect of melatonin on auxin pathway, a KEGG analysis was performed using the DEGs regulated by melatonin treatment (17864 DEGs in root and 21626 DEGs in leaf). First of all, tryptophan metabolism was activated. Second, genes encoding auxin transporters, *AUX1*, were enriched. Third, genes encoding the three key components of auxin signaling, including auxin receptor (*TIR1*), transcriptional repressors (*Aux/IAA*) and auxin response factors (*ARF*), were all enriched. At last, auxin-responsive genes were also enriched, such as *Aux/IAA*, *GH3* and *SAUR*-like genes (**Figure S2**). These auxin-related genes from KEGG analysis were further analyzed to build co-expression network in each treatment, using a pearson correlation method (**Table S4**). Both in the network of melatonin and IAA treatments, the correlations between genes were mainly positive (red edges). Four groups of genes related to tryptophan metabolism, such as *AMI1*, *CYP79B2*, *YUC2* and *YUC4*, were located in the center of the network (center genes). In the network of IAA treatment, the correlations between *YUC4* genes and *SAUR*-like genes formed the core correlations (the ring-like edges in the middle). While in the network of melatonin treatment, the situation was more complicated. All of the four groups of center genes formed such ring-like correlations with *SAUR*-like genes (**Figure 3**). In a word, result of KEGG analysis tells that melatonin treatment gives rise to enrichment of the whole auxin pathway both in root and leaf, while the co-expression network tells that the regulation of melatonin on auxin pathway is more complex than IAA itself.

### Effect of melatonin or IAA treatment on auxin biosynthesis genes

**Figure 4:**
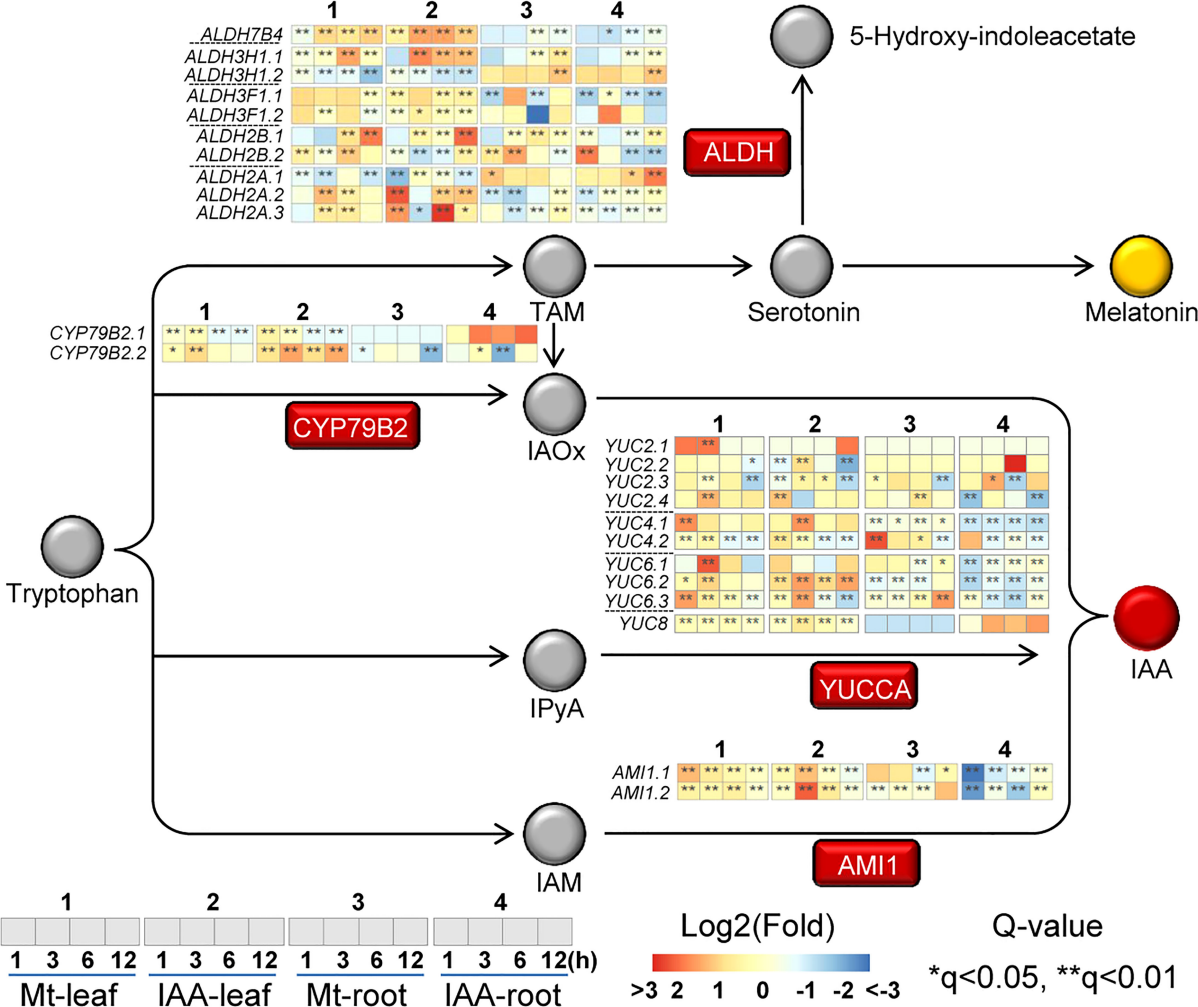
Expression changes of genes related to auxin biosynthesis. The rectangles indicate the enzymes related to melatonin/auxin biosynthesis pathway, while the circles indicate the precursors/metabolites. Italic indicates the gene that may encode the enzyme. Heat-maps indicate expression changes at a time point, while asterisks indicate significant change, compared to the untreated samples (0 h). Asterisks indicate significant differences (*q < 0.05 and **q < 0.01) compared to the untreated samples (0 h).

Because IAA is derived from tryptophan, the activation of tryptophan metabolism suggests that IAA biosynthesis is increased. Thus, we took a deep look at the expression changes of genes in this process. In the four Trp-dependent IAA biosynthesis pathways, three of them had gene expression changes (**Figure 4**) (Mano and Nemoto, 2012). The IAOX (indole-3-acetaldoxime) pathway is activated, for expression of *CYP79B2* was significantly induced by both melatonin and IAA treatments. In the IAM (indole-3-acetamide) pathway, *AMI1* genes were induced by melatonin and IAA treatments in leaf, but were repressed by IAA in root. The most changed pathway is the IPA (indole-3-pyruvic acid) pathway, in which a lot of *YUCCA* genes were affected and their expression patterns were various. Most *YUCCA* genes were induced faster in leaf than in root. In leaf, they reached peak value at 1^st^ or 3^rd^ h, whereas in root some of them were up-regulated until 12^th^ h. In the tryptamine (TAM) pathway melatonin and IAA share some intersection (Kang et al., 2007; Mukherjee, 2018). We observed that several *ALDH* genes, which may be related to the catalysis of serotonin to 5-hydroxy-indoleacetate (Lenchner and Santos, 2022), were up-regulated by both melatonin and IAA (**Figure 4**). This result suggested that the endogenous melatonin biosynthesis may be impacted, for the precursor of melatonin, serotonin, may be catalyzed to other metabolite. To summarize, exogenous melatonin could induce *CYP79B2, AMI1* and several *YUCCA* genes, which also suggests that melatonin treatment may have the ability to trigger auxin biosynthesis.

### Melatonin activates expression levels of genes in auxin signaling pathway

**Figure 5:**
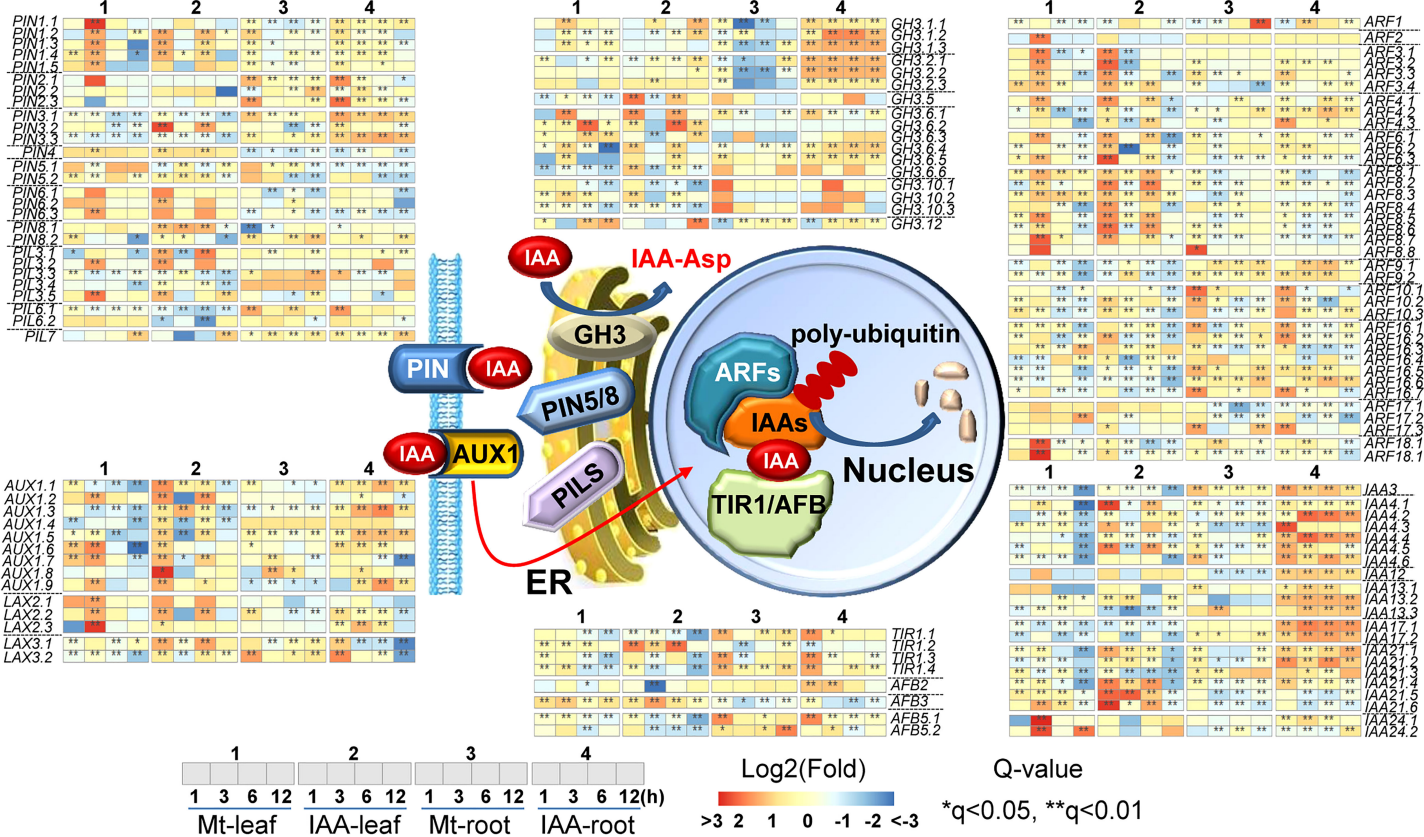
Expression changes of genes related to auxin transportation and signaling pathway. The middle part indicates models of IAA transportation and signaling pathway. Non-italics indicate the enzymes/proteins that catalyze the molecular reaction, while italics indicate the genes which may encode the corresponding proteins. Heat-maps indicate expression changes at a time point, while asterisks indicate significant change, compared to the untreated samples (0 h). Asterisks indicate significant differences (*q < 0.05 and **q < 0.01) compared to the untreated samples (0 h).

In the process of auxin transport, there were 14 *AUX1/LAX* (like *AUX1*) genes and 27 *PIN/PIL* (*PIN*-like) genes, of which expression levels were influenced by melatonin as well as by IAA. In the process of sensing auxin, 8 *TIR1/AFB* genes, 21 *Aux/IAA* (IAAs) genes and 37 *ARF*s were regulated by melatonin or IAA treatment. In the process of auxin homeostasis, 17 *GH3*-like genes were changed in their expression levels (**Figure 5**). As for auxin-responsive genes, the number of regulated *SAUR*-like genes rose to 67 in root and 161 in leaf (**Figure S3**).

To be more specifically, *TIR1/AFB* genes including *TIR1.2*, *TIR1.3*, *TIR1.4*, *AFB3* and *AFB5* were up-regulated. *TIR1.1* was up-regulated in root but not in leaf. For *AUX/LAX* genes, the majority of them were induced by melatonin and IAA treatments. Three genes, *AUX1.1*, *AUX1.3* and *AUX1.4* were only enhanced by IAA treatment. For *PIN/PIL* genes, their expression patterns in melatonin and IAA treatments were very similar. Specifically, *PIN1* genes were up-regulated by the two molecules both in leaf and root; *PIN2* genes were up-regulated by the two molecules in root but not in leaf; *PIN4*, *PIN5* and *PIN6* were induced in leaf but repressed in root by the two molecules; *PIN3* and *PIL3* showed some expression fluctuation in leaf but were mainly induced in root. Most of the *Aux/IAAs*, *GH3*, *ARF* and *SAUR*-like genes were regulated by the two molecules similarly. The up-regulation of *Aux/IAAs*, *ARF* and *GH3* genes was higher under IAA treatment than under melatonin treatment, while the changes of *SAUR*-like genes were very much the same in the two treatments (**Figure S3**). To sum it up, this is a full spectrum response in auxin signaling pathway. Except that few genes, such as *AUX1.1*, *AUX1.3* and *AUX1.4*, had converse expression patterns between melatonin and IAA treatments. The expression tendencies of most genes are very much alike in both melatonin and IAA samples (**Figure 5**). The above results suggest that melatonin treatment could activate the entire auxin signaling pathway.

### Gene expression analysis using quantitative RT-PCR

**Figure 6:**
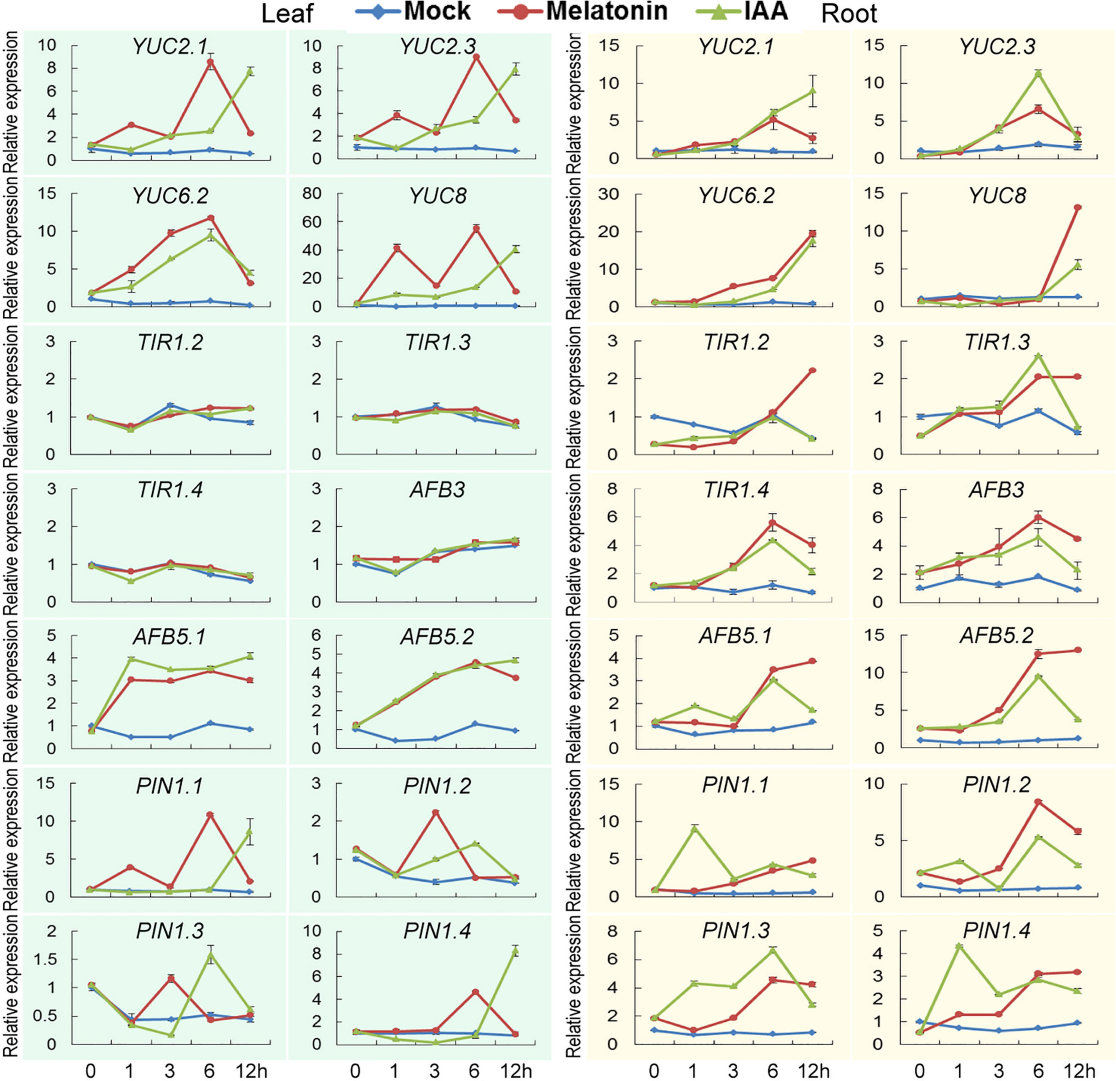
Verification of transcriptome analysis using quantitative PCR. Expression patterns of genes related to auxin biosynthesis and signaling pathways. Error bars indicate SD (n=3).

To verify the results of transcriptome data, a completely independent treatment was performed. Several *YUCCA*, *TIR1*, *AFB* and *PIN1* genes were tested for their expression patterns using quantitative RT-PCR. Besides, a mock treatment was also introduced to observe the expression rhythm of these genes in day time. Under mock treatment, expression of these genes fluctuated very slightly. While under melatonin and IAA treatments, except that three *TIR1* and *AFB3* genes were not induced significantly in leaf, these genes were highly induced. Some of them reached the highest expression levels at certain time points and began to drop, such as *AFB5.2, PIN1.2* and *PIN1.3*, while expression of some genes continued to increase, such as *YUC6.2* and *YUC8* in root (**Figure 6**; **Table S5**). In detail, the expression patterns of these genes in the current treatments were not exactly the same as the transcriptome results, if we compared specific genes at each time point. But in general, the two experiments had something in common. We could see from both transcriptome and qPCR analysis that genes related to auxin biosynthesis and signaling pathways were triggered by both melatonin and IAA treatments.

### DR5 promoter mediated GUS staining suggests melatonin treatment increases auxin content

**Figure 7:**
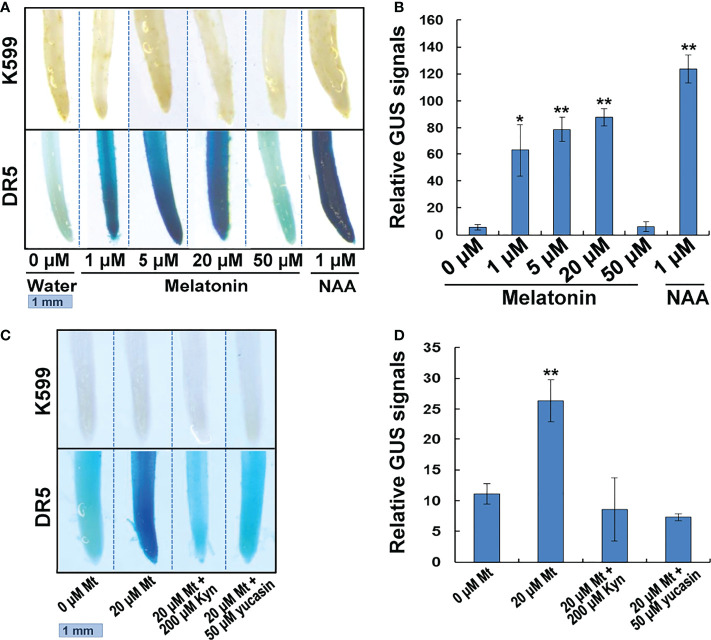
GUS staining of melatonin or NAA treated transgenic soybean roots. **(A)** GUS staining analysis. Transgenic hair roots transformed with DR5:*GUS* gene were treated with melatonin or NAA for 24h. **(B)** Quantitative GUS signals. Relative GUS signals = (Blue light intensity of DR5 roots)/(Blue light intensity of K599 roots). Three roots of each concentration were measured. Value indicates means ± SD (n=3). Asterisks indicate significant differences (*P < 0.05 and **P < 0.01) compared to that of untreated roots (0 μM). **(C)** GUS staining. Transgenic hair roots transformed with DR5:*GUS* gene were treated with melatonin and IAA biosynthesis inhibitor for 24h. **(D)** Quantitative GUS signals. Value indicates means ± SD (n=3). Asterisks indicate significant differences (**P < 0.01) compared to that of untreated roots (0 μM).

DR5 is a synthetic DNA element with great auxin-responsive activity (Ulmasov et al., 1997; Chen et al., 2013). We transformed the DR5-driven *GUS* gene into the soybean hairy roots to test the possible change of auxin response after melatonin treatment. The DR5:*GUS* transgenic hairy roots were treated with different concentrations of melatonin for 24 h. NAA treatment was also performed as a positive control experiment and hairy roots that generated by empty K599 strain were used as a negative control. The results showed that the negative control hairy roots (K599) could not be stained with or without treatment. The water-treated DR5 transgenic roots only had very weak GUS staining. After melatonin or NAA treatment, the intensity of GUS staining remarkably increased. However, when the concentration of melatonin increased to 50 μM, the intensity of GUS staining dropped to the level of water-treated roots (**Figure 7A**). Quantitative analysis showed that melatonin treatment had a dosage effect on DR5:GUS activity, but the induction was not as high as that by NAA treatment (**Figure 7B**). These results indicate that melatonin can activate auxin response.

We further used two kinds of IAA synthesis inhibitors, L-kynurenine (Kyn) and 5–(4–chlorophenyl)-4H-1,2,4–triazole-3–thiol (yucasin), to treat the DR5:*GUS* transgenic hairy roots to see if the two inhibitors could block the auxin response promoted by melatonin treatment. Kyn is an inhibitor of TAA1, which is responsible for the conversion of tryptophan into IPyA (He et al., 2011). Yucasin, is an inhibitor of YUCCA enzymes, which catalyze the conversion of IPyA into IAA (Nishimura et al., 2014). While the 20 μM melatonin significantly enhanced DR5:GUS activity in transgenic hairy roots, treatment with 200 μM Kyn or 50 μM yucasin disrupted this promotive effect. The DR5:GUS activity dropped to the level of untreated hairy roots (**Figures 7C, D**). These results indicate that melatonin activates auxin response through auxin biosynthesis pathway.

### Gene ontology analysis reveals the difference between melatonin and IAA treatment

**Figure 8:**
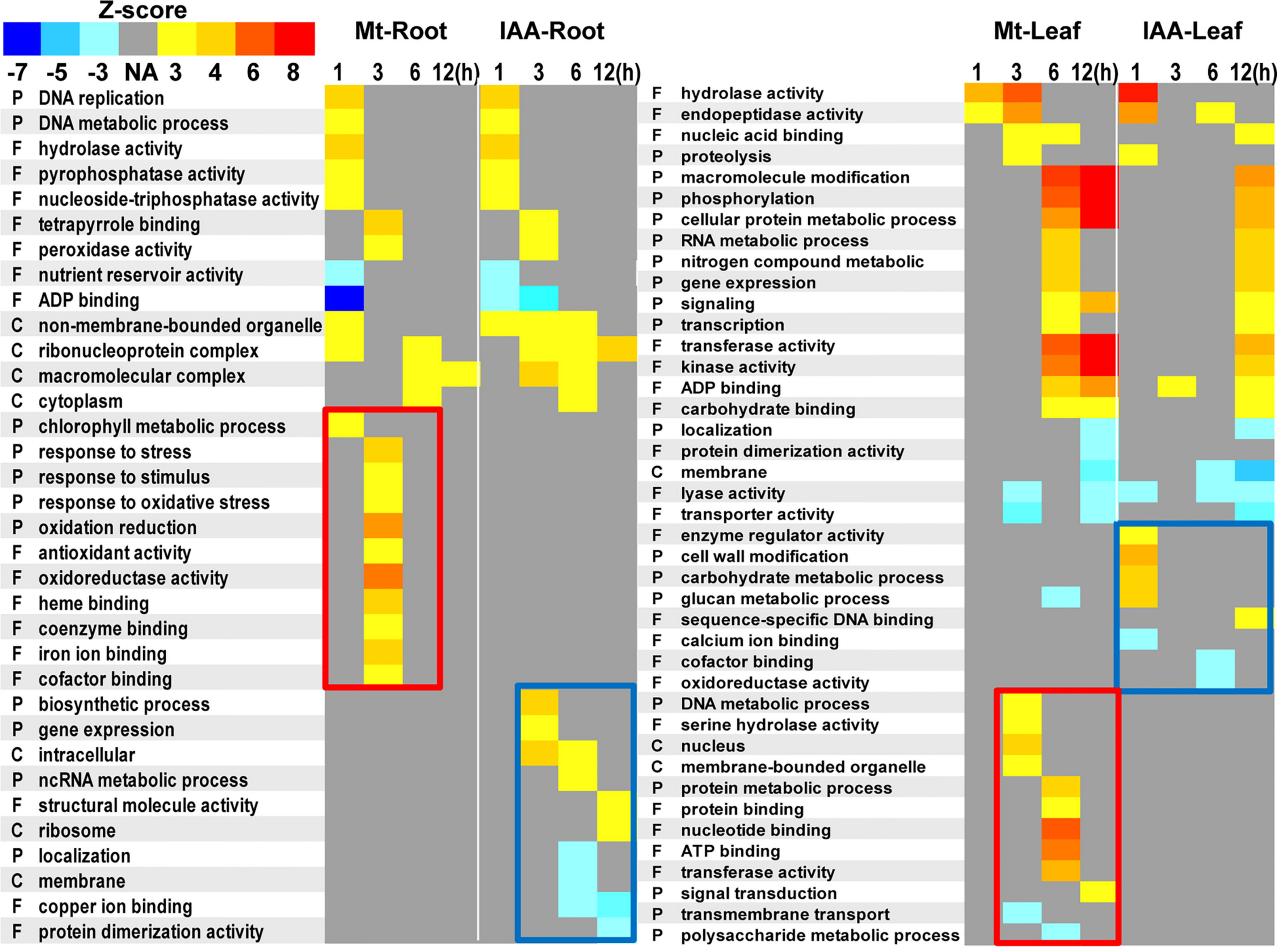
Gene ontology terms enriched in melatonin or auxin treated roots and leaves. DEGs of root or leaf samples were analyzed using agriGO. Statistical significance was evaluated using a Z-score method. P, biological processes; F, molecular functions; C, cellular components. Yellow to red colors indicate up-regulation of the GO terms, while cyan to blue colors indicate down-regulation of the GO terms. Red rectangles indicate specific enrichment in melatonin treatment, while blue rectangles indicate specific enrichment in IAA treatment.

We performed a Gene ontology analysis using the 23529 DEGs in root and the 26481 DEGs in leaf. Results showed that melatonin and IAA treatments had a lot in common. There were numbers of GO terms, which were simultaneously up-regulated or down-regulated by the two molecules (**Figure 8**, unframed GO terms). It is worth mentioning that such GO analysis also discloses the unique characteristics of each molecule. For melatonin treatment, exogenous melatonin up-regulated the GO terms that were related to stress response, especially oxidative stress. While in leaf, melatonin up-regulated GO terms that were involved in metabolic or catalytic process (**Figure 8**, red rectangles). For IAA treatment, the GO terms relating to growth were enriched, including biosynthetic process, gene expression, cell wall modification and energy metabolic processes (**Figure 8**, blue rectangles).

## Discussion

Melatonin is an important bio-active molecule in living organisms. The fact that melatonin has a close correlation with the plant hormone auxin makes melatonin even more fascinating. For example, melatonin has the ability to enhance plant growth, such as promoting root development and enlarging plant size (Arnao and Hernandez-Ruiz, 2007; Wei et al., 2015). These studies suggest that melatonin may play certain auxin-like roles in plants. For more than ten years, scientists have been trying to figure out the relationship between these two chemicals, but their specific relationship still keeps unknown.

Our study demonstrates that melatonin activates several *YUCCA* genes in the IPyA pathway, which is the major biosynthesis pathway of auxin biosynthesis (Mashiguchi et al., 2011). Through enhancing auxin biosynthesis, melatonin also activates the entire auxin transportation and response pathways (**Figure S2**). The hypothesis is explained from the follow aspects.

First of all, 60% of the DEGs were commonly regulated by melatonin and IAA treatments, and such ratio was very high (**Figure 1**). Almost all of these common genes were able to be clustered according to their expression patterns. Both in root and leaf, the common genes of melatonin and IAA treatments showed great similarities, suggesting the close connection between melatonin and IAA (**Figure 2**). Second, melatonin treatment could enhance expression of auxin biosynthetic genes. Melatonin does not equally enhance the four pathways of auxin biosynthesis. YUCCA proteins are responsible for the conversion of IPyA to IAA (Yamamoto et al., 2007; Mashiguchi et al., 2011). *YUCCA* genes in the IPyA pathway were mostly influenced by melatonin (**Figures 4** and **6**). Third, if the amount of IAA was changed, the auxin transportation and perception processes would be affected as a result (Fig 5). Auxin transport contains two kinds of auxin carriers, the AUX/LAX influx carriers and the PIN/PIL efflux carriers (Kramer, 2004; Peret et al., 2012). Situations were different between some of the *AUX/LAX* genes and *PIN/PIL* genes. No matter up-regulated or down-regulated, *PIN/PIL* genes were regulated by melatonin and IAA treatments in the same direction. But for some *AUX1* genes, including *AUX1.1*, *AUX1.3* and *AUX1.4*, they were up-regulated by IAA but down-regulated by melatonin (**Figure 5**). PIN/PIL proteins are responsible for intracellular auxin homeostasis, expression of *PIN/PIL* are influenced by cellular auxin content (Cazzonelli et al., 2013; Adamowski and Friml, 2015). Other group also found that melatonin influenced expressions of *PIN* genes (Wen et al., 2016). In our experiment, *PIN/PIL* genes in leaf were up-regulated by both melatonin and IAA (**Figures 5** and **6**). These expression patterns match the patterns of *YUCCA* genes (**Figure 4**). Because both melatonin and IAA treatments enhanced cellular auxin content, *PIN/PIL* genes were regulated similarly under the two treatments. We also observed that AUX/LAX proteins are different auxin transporters from PIN/PIL proteins. It is reported that AUX1 has auxin uptake activity (Peret et al., 2012). There was no exogenous applied IAA in melatonin solution, so expression of these *AUX1* genes were not as high as that promoted by IAA (**Figure 5**).

The above transcriptome analysis supports our conclusion very well. And at last, a DR5:GUS staining provides the strongest evidence. DR5:GUS activity was increased significantly by melatonin treatment. But if we added inhibitors of IAA biosynthesis, such as L-Kyn and yucasin to the melatonin treatment, the above increasing effect disappeared. These results indicate that IAA biosynthesis is required for the melatonin-activated DR5:GUS activity. Besides, the GUS staining experiment also uncovered the ‘concentration effects’. Melatonin treatment promotes IAA content in a dosage manner. But if the concentration was too high, for instance, when concentration was increased to 50 μM, the promoting role disappeared (**Figure 7**). This result was consistent with the previous studies that coating seeds with 50 or 100 μM melatonin had stimulating effect on soybean growth. Considering that melatonin was applied in a slow-releasing method using seed-coating-reagent, the actual melatonin amount was much lower than 50 or 100 μM (Wei et al., 2015). Similarly, in the earliest work that compared the effect of melatonin with auxin on promoting lupin roots, the maximal effect was observed at a concentration of 10 μM melatonin (Arnao and Hernandez-Ruiz, 2007). However, some studies demonstrated that melatonin had no effect or negative effect on auxin response in Arabidopsis (Pelagio-Flores et al., 2012; Weeda et al., 2014; Wang et al., 2016). The above results suggest that the effect of melatonin on auxin response occurs differently between brassicaceae and fabaceae plants.

The puzzle of the auxin-like feature of melatonin has been confusing to us for years, not only because brassicaceae and fabaceae plants performed differently, but also because transgene events had the negative results (**Table S1**). Two studies provided the evidence that over-expressing *AANAT* (the corresponding gene in plant is *SNAT*) or *ASMT* gene could enhanced melatonin content but decreased endogenous IAA content in transgenic plants (Wang et al., 2014; Zuo et al., 2014). We suggest a trade-off hypothesis to explain these phenomena in soybean or other plants that had similar effects (**Figure S4A**). If large amount of tryptophan was recruited to produce melatonin, the biosynthesis of IAA would be reduced (**Figure S4B**). Conversely, if melatonin biosynthesis was somehow repressed, tryptophan would go to IAA pathway. For exogenous melatonin treatment, melatonin is taken up by plant roots from the environment (Tan et al., 2012). In such circumstance, the need for melatonin biosynthesis would not be as necessary as it used to be. So we suppose that the metabolic pathway from tryptophan to IAA is then activated (**Figure S4C**).

**Figure 9:**
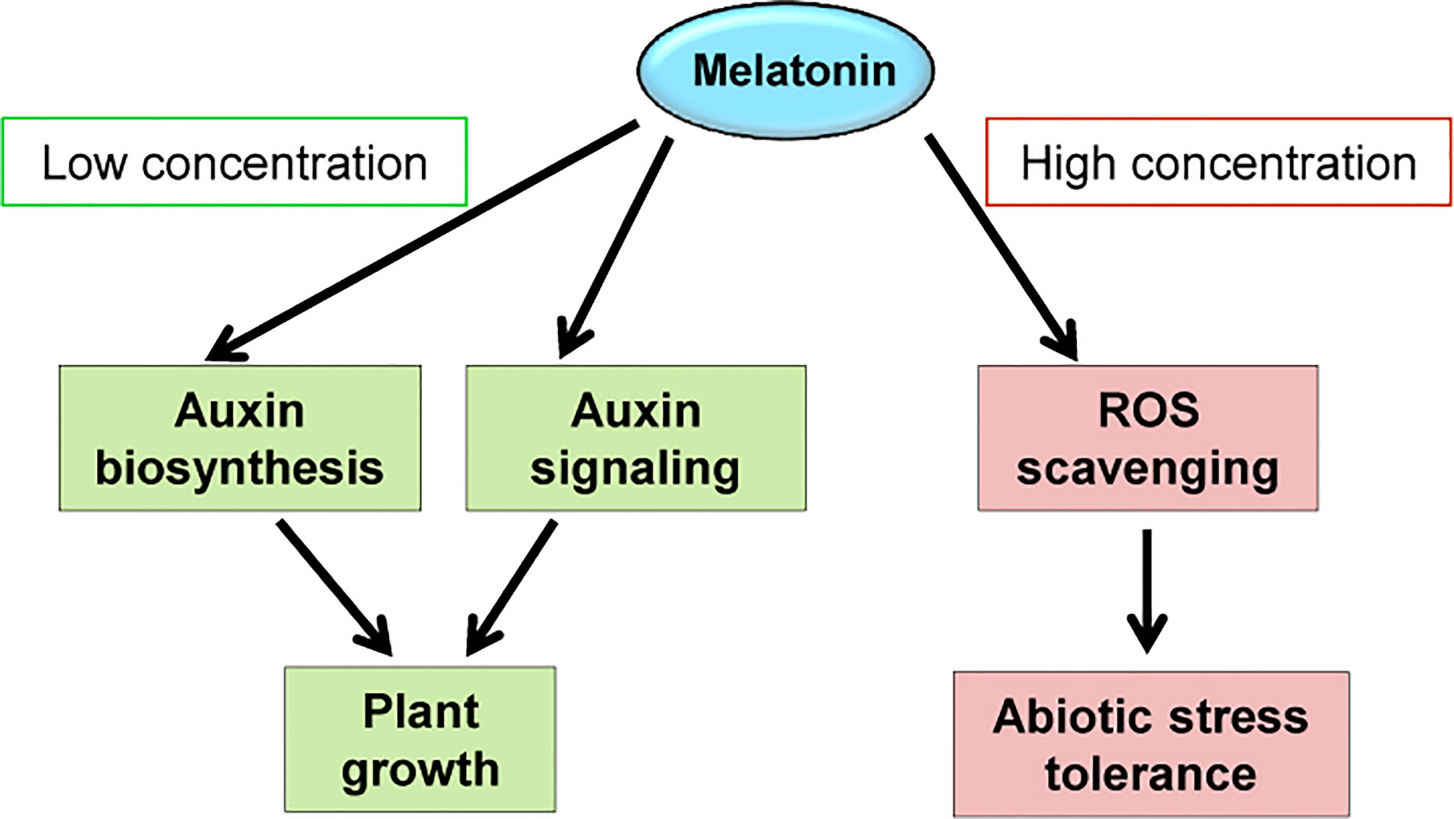
Possible working models of melatonin. The working models demonstrate the molecular changes that caused by exogenously applied melatonin.

Together, we propose that melatonin and IAA may regulate similar sets of genes for functions, and melatonin may promote auxin responses at least partially through activation of auxin biosynthesis and/or signaling pathway. This knowledge would facilitate application of melatonin in agriculture for yield promotion. As for soybean or plants like soybean, low concentration of melatonin could induce auxin biosynthesis and signaling pathways and therefore promote plant growth. To help plants resist against various stresses, continuous melatonin supply is needed to scavenge ROS, thus the concentration need to be higher than normal conditions (**Figure 9**). We establish a method with the best effect on both needs, which is coating seeds at a relative high melatonin concentration using slow-released reagent (Wei et al., 2015).

## Data availability statement

The raw data of RNA-seq reported in this paper have been deposited in the Genome Sequence Archive in Beijing Institute of Genomics (BIG), Chinese Academy of Sciences (accession numbers: CRA002667), http://bigd.big.ac.cn/gsa.

## Author contributions

WW performed experiments and drafted the initial manuscript; WW, J-JT and C-CY conducted data analysis; W-KZ, S-YC and J-SZ, conceived projects, designed experiments and wrote the paper. All authors contributed to the article and approved the submitted version.
