# Supplementary material for: Melatonin regulates gene expressions through activating auxin synthesis and signaling pathways
Source: Front Plant Sci. 2022 Dec 13;13:1057993. doi: 10.3389/fpls.2022.1057993 (PMC9792792; doi:10.3389/fpls.2022.1057993)
Supplement: Supplementary file 1 [file Table_1.docx]

Table S1. The effects of melatonin on IAA synthesis or IAA-responsive genes.

| **Plant** | **Method** | **Mt content** | **Measurement** | **Effect** | **Reference** |
| --- | --- | --- | --- | --- | --- |
| ***Brassica juncea*** | **Exogenous Mt** | **0.01-0.5 μM** | **IAA content** | **increased** | **Chen *et al*., 2009** |
| **Arabidopsis** | **Exogenous Mt** | **150-600 μM** | **DR5*:uidA*** | **unchanged** | **Pelagio-Flores *et al*.,**  **2012** |
| **Arabidopsis** | **Exogenous Mt** | **50-500 μM** | **DR5*:GUS*** | **unchanged** | **Koyama *et al*., 2013** |
| **Tomato** | **Transgene** | **2-5 fold** | **IAA content** | **decreased** | **Wang *et al*., 2014** |
| **Arabidopsis** | **Exogenous Mt** | **1 mM** | **IAA-responsive genes** | **down-regulated** | **Weeda *et al*., 2014** |
| **Arabidopsis** | **Transgene** | **4 fold** | **IAA content** | **decreased** | **Zuo *et al*., 2014** |
| **Arabidopsis** | **Exogenous Mt** | **600 μM** | **IAA responsive genes** | **down-regulated** | **Wang *et al*., 2016** |
| **Tomato** | **Exogenous Mt** | **50 μM** | **IAA content** | **increased** | **Wen *et al*., 2016** |
