# Supplementary material for: Melatonin regulates gene expressions through activating auxin synthesis and signaling pathways
Source: Front Plant Sci. 2022 Dec 13;13:1057993. doi: 10.3389/fpls.2022.1057993 (PMC9792792; doi:10.3389/fpls.2022.1057993)
Supplement: Supplementary file 3 [file DataSheet_2.pdf]

Melatonin regulates gene expressions through activating auxin synthesis and signaling pathways

Wei Wei, Jian-Jun Tao, Cui-Cui Yin Cui-Cui Yin, Shou-Yi Chen,
Jin-Song Zhang and Wan-Ke Zhang

Correspondence Authors: **Wan-Ke Zhang, Shou-Yi Chen and Jin-Song Zhang**

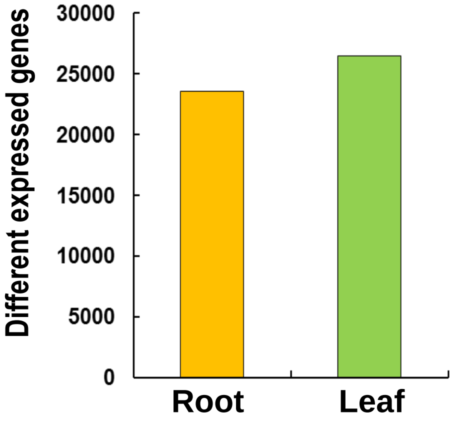

Figure S1. DEG analysis of transcriptome data.

Total DEGs (different expressing genes) in soybean roots and leaves. Genes that had a two-fold increase or a 50% decrease in at least one time point against untreated samples (0 h) were defined as differentially expressed genes (DEGs). Melatonin and auxin samples were counted together.

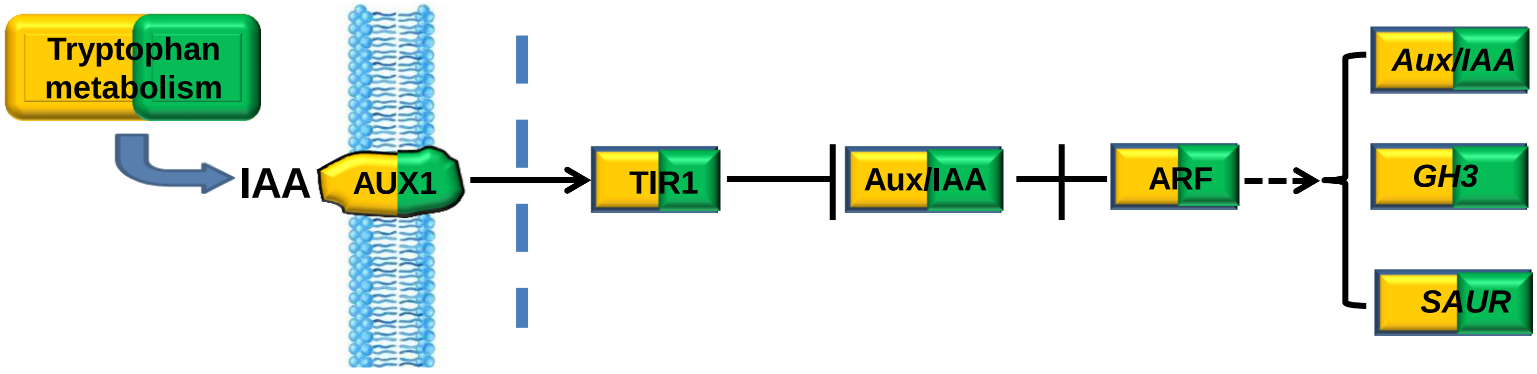

Figure S2. Melatonin treatment enriched DEGs in auxin biosynthesis and signaling pathways.

A KEGG analysis was performed using the DEGs in melatonin treatment. Yellow color indicates enrichments in root, while green color indicates enrichments in leaf.

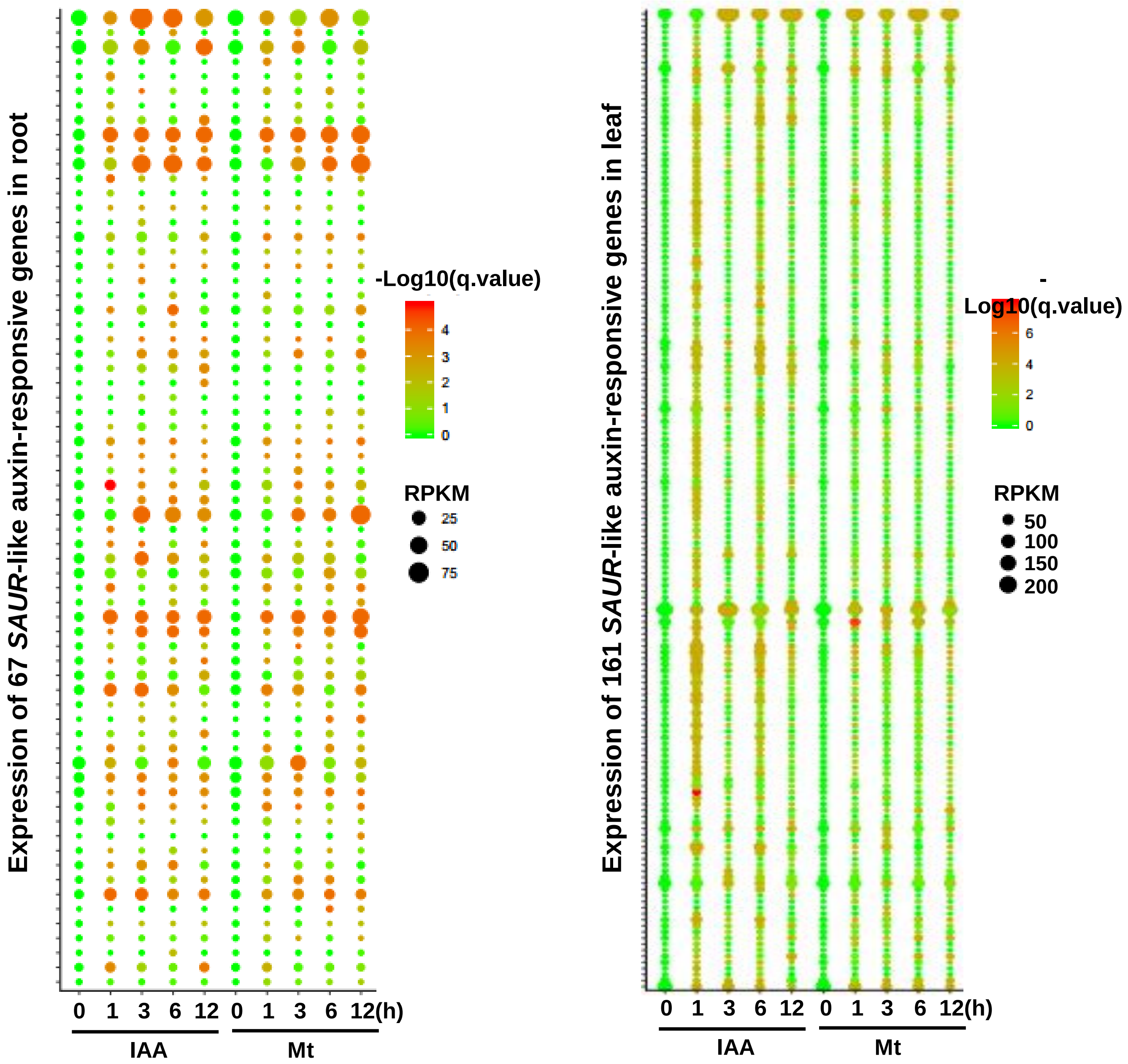

Figure S3. Bubble heat-map of *SAUR-like* auxin-responsive genes.

Size of the bubbles indicates RPKM data of genes. Red color indicates significant change of a time point, compared to the time point of 0 hour.

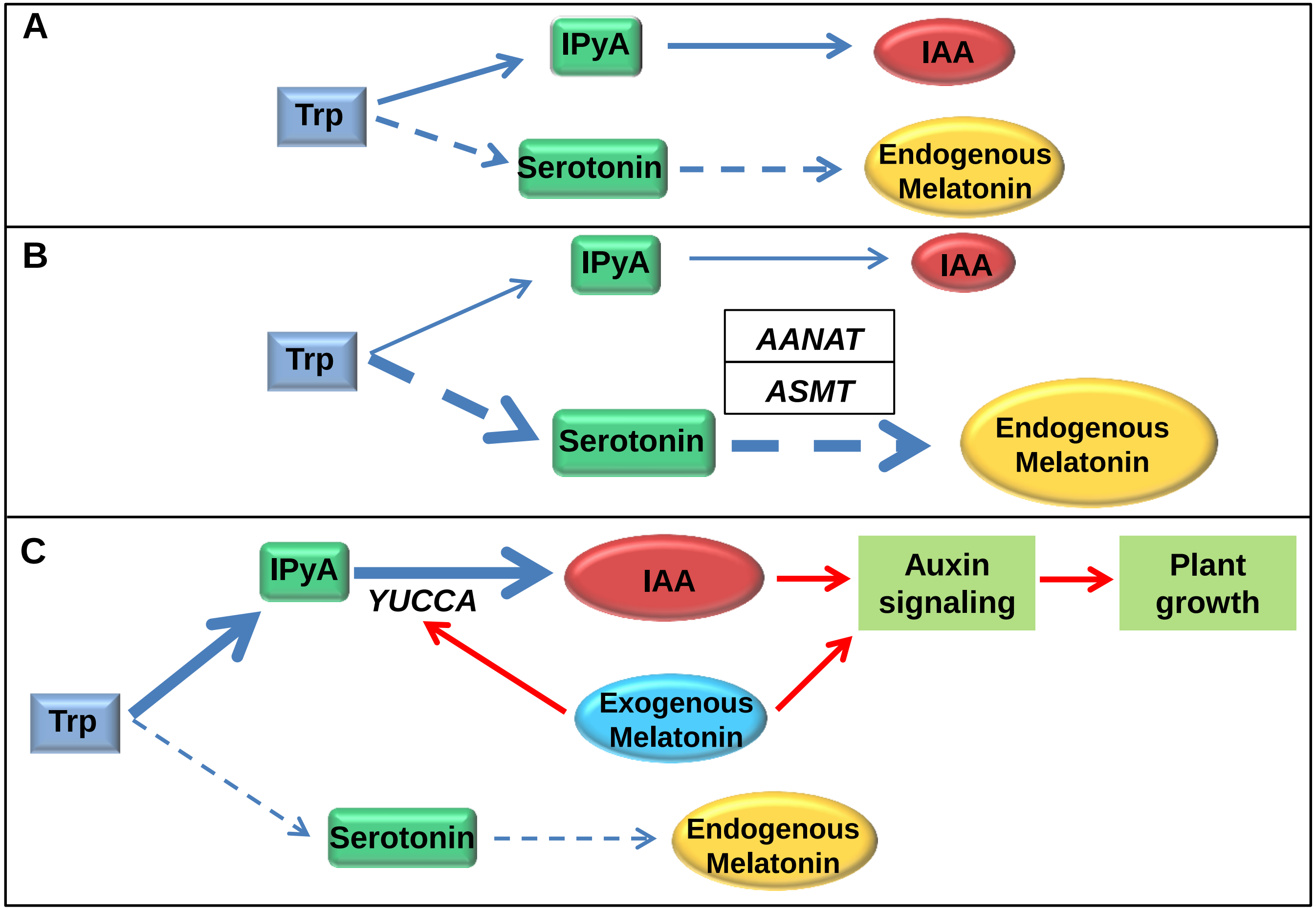

Figure S4. Tryptophan balance between melatonin and auxin biosynthesis pathways.

(A) The metabolic balance between IAA and melatonin biosynthesis under normal conditions. Blue arrows indicate metabolic processes, in which dotted lines indicate omissions of certain catalysis steps. (B) The effect of transgenic events towards melatonin and auxin biosynthesis. Framed italics indicates transgenic events. This part mainly bases on the work of Wang *et al*., 2014 and Zuo *et al*., 2014. (C) The effect of exogenous applied melatonin towards melatonin and auxin biosynthesis. This part mainly bases on the analysis in this study, including up-regulation of many *YUCCA* genes and IAA signaling genes by melatonin treatment. Red arrows indicate that the expression levels of these genes are up-regulated or the processes are enhanced.
